# Recent advances in PLGA polymer nanocarriers for ovarian cancer therapy

**DOI:** 10.3389/fonc.2025.1526718

**Published:** 2025-03-24

**Authors:** Tingjing You, Shengmin Zhang

**Affiliations:** ^1^ Ningbo University, Ningbo, China; ^2^ Department of Ultrasound Medicine, The First Affiliated Hospital of Ningbo University, Ningbo, China

**Keywords:** polymer, ovarian cancer, PLGA, combination chemotherapy, nanocarrier

## Abstract

Ovarian cancer (OC) is the most lethal gynecologic malignancy worldwide, and early diagnosis and effective treatment have been the focus of research in this field. It is because of its late diagnosis, acquired resistance mechanisms, and systemic toxicity of chemotherapeutic agents that the treatment of ovarian cancer is challenging. Combination chemotherapy can potentially improve therapeutic efficacy by activating multiple downstream pathways to overcome resistance and reduce the required dose. In recent years, PLGA-lipid hybrid nanoparticles have demonstrated their potential as an emerging drug delivery system for treating ovarian cancer. PLGA (poly (lactic-co-glycolic acid) has become a highly sought-after biomaterial for the clinical translation of adjustable drug delivery regimens due to its biodegradability, biocompatibility, and multifunctionality, coupled with controlled drug release, which can effectively overcome multidrug resistance and improve the efficiency of chemotherapy. Combination therapies are gradually becoming an ideal alternative to traditional drug formulations. The application of nanoparticles not only improves the therapeutic effect but also reduces the side effects, which provides strong support for personalized precision medicine. We review polymeric nanoparticle carriers for drug combinations used in the treatment of ovarian cancer, particularly the combination of paclitaxel analogs (commonly used first-line therapy for ovarian cancer) with other small molecule therapeutic agents and cavitation combination therapy under ultrasound targeting (**Figure 1**). The elucidation of these issues will provide a theoretical basis for future exploration of novel NNDDS targeting GRPR for anti-OC therapy. This review presents research on recent advances in PLGA polymer nanoparticles in ovarian cancer, focusing on the use of PLGA degradable microspheres for loading chemotherapeutic agents and ultrasound combination therapy.

## 1 Introduction

Ovarian cancer is the leading cause of gynecologic malignancies in women. Ovarian cancer is asymptomatic in its early stages, leading to advanced stages and lower survival rates when patients are diagnosed. According to data released by the International Agency for Research on Cancer (IARC), there were 313,959 new cases of OC globally in 2020, projected to rise to 348,000 by 2025 (1). The incidence of OC is rising at an alarming rate in many parts of the world, and it is estimated that 19,710 new cases of ovarian cancer will be diagnosed in 2023 in the United States (2), OC remains the deadliest gynecologic malignancy (3). Although cytoreductive surgery is the treatment of choice for ovarian cancer, chemotherapy alone or in combination with surgery is usually used to eradicate unresected lesions (4).In contrast, conventional chemotherapeutic agents often bring significant side effects, such as vomiting, emesis, and alopecia, which may affect patients’ quality of life and treatment dependence. For patients with advanced stage III-IV disease, whose tumors are unlikely to be completely reduced to no residual disease (R0) or who are not suitable for surgery, neoadjuvant chemotherapy (NACT) is an increasingly important potential treatment option for these patients (5). Among them, platinum-containing drugs and paclitaxel are part of the standard NACT regimen, which is widely used in the advanced treatment of ovarian cancer. In recent years, the use of PLGA in targeted drug delivery systems has increased dramatically due to its use as a biodegradable, non-toxic, non-immunogenic polymer. Several studies have combined PLGA with chemotherapeutic drugs, where PLGA nanoparticles can encapsulate chemotherapeutic drugs, protect them from enzymatic and metabolic effects *in vivo*, and increase their stability, thus extending their half-life *in vivo*. The degradation products of PLGA are lactic acid and glycolic acid, both of which are endogenous and can be simply metabolized by the body, and are expected to have negligible systemic toxicity (6).PLGA nanoparticles have been successfully used to encapsulate various cancer-related drugs and their successful delivery *in vivo*. Cancer-related drugs paclitaxel, Adriamycin, 5-fluorouracil, 9-nitro-camptothecin, cisplatin, Treprostinil, dexamethasone, flavonoids, etc. have been successfully encapsulated on PLGA nanoparticles. Biodegradable nanoparticles are frequently used to improve the therapeutic value of various water-soluble/insoluble drugs and bioactive molecules by enhancing bioavailability, solubility, and retention time (7). Compared to traditional drug delivery methods, nanoscale materials can induce drug delivery to specific targets in the body more accurately, significantly increasing drug concentrations in target tissues while reducing systemic toxicity. In addition, these materials are dosed to extend the half-life of the drug in the bloodstream, improving bioavailability and thus enhancing overall therapeutic efficacy (8). These nanoparticle drug formulations reduce the cost and risk of toxicity for patients and are a good solution for treating ovarian cancer patients with poor prognoses due to the side effects of chemotherapy drugs (9).

**Figure 1:**
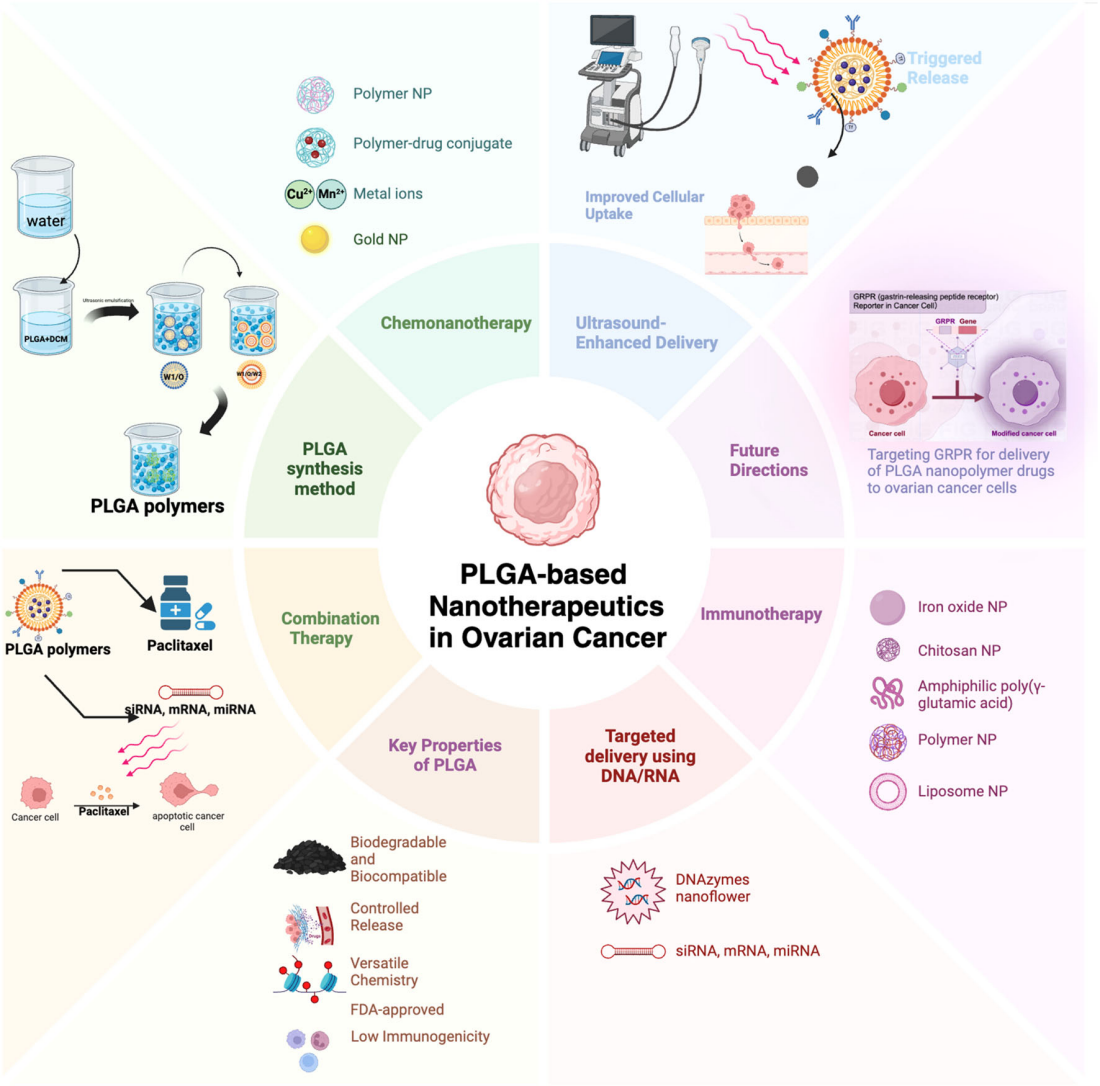
Schematic representation of PLGA-based nanotherapeutics for ovarian cancer.

## 2 Polylactic acid-glycolic acid copolymer

Polymeric nanoparticles are submicron-sized colloidal particles that effectively encapsulate or bind drugs (10).In the field of pharmaceutical particles, a variety of polymers have been used, such as chitosan, poly (lactic acid) and poly(1-caprolactone). One of the most widely used polymers is poly(lactic-co-ethanolic acid) (PLGA), a copolymer of lactic and ethanoic acids, which is particularly suitable for clinical and biological applications due to its low toxicity, low immunogenicity, biocompatibility, and biodegradability. PLGA is a class of FDA-approved, synthetic, biodegradable, biocompatible, and nontoxic polymers substituted for biodegradable, non-toxic, biologically non-toxic, and biocompatible and is approved by the FDA (11). Polymeric NPs(nanoparticles) offer some specific advantages over liposomes. For example, NPs contribute to drug/protein stability and have pharmacologically active agent-controlled release properties. Several examples of PLGA polymer nanocarrier interactions for therapeutic use are given in this paper.

### 2.1 Preparation and application of polymer nanoparticles

Several methods are available for synthesizing PNPs (12), Depending on whether the formulation requires a polymerization reaction or is made directly from macromolecules or preformed polymers, these methods can be divided into two main categories (13). Traditionally, NPs are mainly prepared by two methods:**(i)** dispersion of preformed polymers: solvent evaporation, spontaneous emulsification/solvent diffusion, salting out/emulsification-diffusion, etc.;**(ii)** polymerization of monomers. Chloroform, dichloromethane, and ethyl acetate are commonly used organic solvents. The use of prefabricated polymers instead of monomers is also constantly being used in applications, where prefabricated polymers are synthesized and then allowed to be dispersed - the emulsification-solvent evaporation method involves two steps. In the first step, the polymer solution needs to be emulsified into an aqueous phase; in the second step, the polymer solvent is evaporated, leading to the precipitation of the polymer in the form of nanospheres. The polymer precipitates in the form of nanospheres, in which the drug is finely dispersed in the polymer matrix network. The solvent is then evaporated through temperature elevation under pressure or continuous stirring (14). In addition to the conventional methods mentioned above, other techniques for preparing polymer nanoparticles are widely used, such as spray drying and soft lithography particle molding. Taking spray drying as an example, polymer nanoparticles consisting of polyglutamic acid and polylysine with a particle size of about 400-500 nm were first prepared using spray drying (15). Subsequently, these polymer nanoparticles were dispersed into a dichloromethane solution containing lipids (e.g., tripalmitate and cetyl alcohol) to form a lipid-polymer suspension. Next, the suspension was spray-dried twice to finally prepare polymer nanoparticles with uniform particle size and coated by lipids. This approach not only helps to control the size distribution of the nanoparticles but also improves the biocompatibility and stability of the particles through lipid coating, thus providing a more optimized carrier for drug delivery systems. Peracchia et al. (16) prepared diblock Me-PEG-PLA copolymer NPs loaded with 20% and 33% lidocaine by emulsification-solvent evaporation. They confirmed the high density of surface PEG by ESCA. However, the size of the NPs prepared from the block copolymers was twice as large as that of the PLGA NPs. This is due to the increased chain length of PEG. Peracchia et al. (17) also prepared methoxy PEG cyanoacrylate-hexadecyl cyanoacrylate amphiphilic NPs by polymer precipitation or solvent evaporation; the PEG coatings were confirmed by XPS. Several studies have also utilized nanoparticles to deliver proteins and peptides, which are unstable and susceptible to degradation in PLGA due to their hydrophobic and acidic nature (18). Kawashima et al. (19) developed a novel pulmonary drug delivery system for the administration of PLGA nanospheres (400 nm size) coated with physiologically active peptides. The aqueous dispersion of PLGA nanospheres was administered via the lungs of guinea pigs by nebulization and significantly reduced blood glucose levels. Saghir et al. (20) made thymoquinone nanoparticles from a combination of PLGA and poly(vinyl alcohol) (PVA), which is also an anticancer drug that acts through selective antioxidant activity in fibrotic rats. The addition of thymoquinone to PLGA-PVA nanoparticles is a promising formulation to ameliorate bleomycin-induced pulmonary fibrosis by modulating TGF-β1 and IL 10 as well as down-regulating iNOS in lung tissue.

### 2.2 Characterization of PLGA nanoparticles

As a synthetic polymer, PLGA has become one of the most widely used drug delivery vehicles (21), capable of transporting proteins, peptides, bacterial or viral DNA, and a wide range of anti-cancer drugs (22). The physicochemical properties of PLGA can be altered by adjusting the ratio (LA/GA), molecular weight (MW), concentration, and end-groups, thus affecting the encapsulation rate (EE%) and drug release kinetics of PLGA microspheres (23). It was shown that when the ratio of LA to GA was 50:50, the reaction rate was accelerated because the polymer was the most crystalline and hydrophilic at this ratio, which made it easier for water to penetrate the polymer matrix (24). In addition, PLGA polymers with high molecular weights exhibit faster degradation rates due to better structural integrity (25). In addition to modulating the properties of the PLGA itself (26), it is also possible to target ligands, such as antibodies or small molecules, by surface chemical modification.

From this, we list the differences in particle size, encapsulation rate, and release kinetics of microspheres prepared from different PLGAs (**Table 1**).

**Table 1:** Physicochemical characteristics of drug-loaded PLGA microspheres: particle size, encapsulation efficiency, and release kinetics.

Loaded drugs	PLGA	Particle size	Encapsulation efficiency (EE%)	Release kinetics	References
Ciprofloxacin HCl	To 5 ml of acetonitrile were added different amounts of PLGA, i.e., 100, 200, 400 mg	42.3, 59.7 and 62.2 micrometers, respectively.	EE% were 90.0%, 93.8%, and 95.3%, respectively, which were positively correlated with PLGA concentration	Reduced rate of drug release	(27)
Doxorubicin (Dox)	PLGA 50:50 and PLGA 75:25	363.1 and 361.4 nm respectively	EE% of 48.37 and 38.65% respectively	Over 20 days, 70.98% of the entrapped drug was released from PLGA 50:50 nanoparticles, whereas PLGA 75:25 nanoparticles released 62.22% of the drug.	(28)
Doxycycline Hyclate	PLGA 50:50 with different end group, i.e., Purasorb^®^PDLG 5002,/5002 A (MW: 17 KDa), and 5004/5004 A(MW: 44 KDa)	Regardless of the end groups and molecular weight of the PLGAs, their average size is around 1 µm.	Microspheres made from polylactic acid glyoxal (PLGA) with high molecular weight and ester-terminated groups show significantly higher encapsulation efficiency	PDLG 5002 A, 5004 A, 5002, and 5004 had drug release rates of approximately 92%, 89%, 47%, and 43%, respectively, over 42 days.	(29)
Paclitaxel (PTX)	PLGA 50:50, molecular weight: 48,000 Da; characteristic viscosity: 0.53 dL/g	Average diameter less than 250 nanometers	A further increase in the initial drug amount (2.25; 4.5; 6.75 mg) did not result in a significant difference in the encapsulation rate (EE%) when the polymer mass was kept constant (45 mg) and in all cases the EE (%) was higher than 85%.	The initial burst phase of PTX release (up to ≈25% within 1 hour), with the remaining PTX molecules released in a more sustained manner over 15 days. NPs with higher drug loadings (10% and 15% w/w) showed the same trend but with a faster release pattern, with approximately 90% of PTX released within 15 days for 15% PLGA-PTX NPs	(30)

## 3 PLGA nanocarriers in ovarian cancer therapy

To date, there are very few reports on the use of PLGA polymer nanoparticles for ovarian cancer *in vitro* and *in vivo* trials through active tumor targeting, but there are similar applications for highly malignant tumors.PLGA polymer nanoparticles can be coupled to a variety of drugs through simple synthesis, and their stability is superior to that of polymer micelles, which require a higher concentration than a critical micelle to form.

### 3.1 Combination therapy based on paclitaxel PLGA-loaded nano microbubbles

Paclitaxel interferes with the normal function of microtubule breakdown by binding to the beta subunit of microtubule proteins. This drug promotes the polymerization of microtubule proteins, leading to cell death by disrupting the kinetics necessary for cell division. This drug has anti-tumor activity against primary ovarian and breast cancers and is now widely used in chemotherapy for ovarian, prostate, breast, and bladder cancers (31). However, conventional paclitaxel formulations are very limited in clinical application due to the toxic side effects of co-solvents and PTX itself, and nano-formulations are a potential strategy to solve the above dilemma (32), Combinations of paclitaxel analogs into polymeric nanoparticles have also been considered. Encapsulation in nanoparticles significantly improves the pharmacokinetic and pharmacodynamic properties of the drug compared to free paclitaxel. In this context, nanoparticle carriers prepared using lactic acid-glycolic acid copolymer (PLGA) have been approved by the FDA for human applications due to their excellent biocompatibility and low toxicity. The binding mechanism of PTX-PLGA mainly relies on physical encapsulation and hydrophobic interactions. In the fabrication of nanoparticles, PTX is usually co-dissolved with PLGA in an organic solvent by nanoprecipitation or emulsification-solvent evaporation. Upon mixing with an aqueous phase containing a stabilizer, the solvent rapidly diffuses, and the polymer precipitates, resulting in the spontaneous formation of PLGA nanoparticles. Since both PTX and PLGA are hydrophobic, the drug is encapsulated in the polymer matrix and the intermolecular hydrophobic interactions stabilize the PTX molecules. In summary, this combined mechanism provides both a protective reservoir for PTX and a platform for its gradual, targeted release, which has been demonstrated to be more efficacious and cytotoxic *in vitro* studies compared to conventional agents. Currently, nab-PTX has been widely used in the treatment of various tumors in the clinic and is a novel nano-formulation of PTX (33), which is a colloidal suspension of nab-PTX nanoparticles with an average diameter of 130 nm, which avoids solvent-related hypersensitivity reactions, toxicity, and complications and does not require pre-treatment. In addition, albumin effectively promotes PTX accumulation in tumors through interaction with the gp60/caveolin-1 receptor. For example, Zhang et al. found the use of a lipid polymer hybrid nanoparticle platform for the co-delivery of paclitaxel and powdered antipyrine (P-glycoprotein inhibitor). Paclitaxel was bound to PLGA and co-loaded into the lipid polymer nanoparticles with powdered antipyrine via nanoprecipitation. Cytotoxicity was assessed *in vitro* using ovarian cancer cell lines. The co-delivered drugs showed synergistic effects, and they also observed increased intracellular paclitaxel accumulation, which induced apoptosis in A2780/PTX cells (34). Yeongseon et al. (35) successfully developed a hyaluronic acid-labeled poly(d,l-propylene glycol-glycolic acid) nanoparticle (HA-PLGA-NP), which was encapsulated with paclitaxel (PTX) and adhesion plaque kinase (FAK) siRNAs, to attach HA (hyaluronic acid) to the surface of PLGA-NPs, thereby targeting the CD44 receptor on the tumor cells. And delivering the drug to the tumor site via the EPR effect. Delivering the drug to the tumor site through the EPR effect, this approach helps to provide therapeutic benefit by overcoming the chemoresistance often observed in cancer treatment.

### 3.2 PLGA polymer nanoparticles with lipid-based nanoparticle delivery system

The nano drug delivery system has been widely validated in the anti-tumor application of natural drugs. The system not only significantly improves the targeting delivery efficiency of drugs in ovarian cancer treatment, but also achieves precise control of drug release and prolongs the blood circulation time of the drugs, thus maximizing their anti-tumor effects (36). Lipid-based nano drug delivery systems, which mainly include liposomes and solid lipid nanoparticles (SLNs), are one of the most widely used lipid-based drug delivery carriers, precisely because lipids have hydrophilic heads and hydrophobic tails, which can be used to transport hydrophilic and hydrophobic drugs and have a wide range of applications (37). Their membrane-like composition facilitates rapid cellular uptake and release of drugs. However, lipid systems are sometimes limited in their drug-loading capacity and may be less stable during circulation. PLGA polymers Nanoparticles are the most widely used and effective polymeric drug delivery systems, with adjustable degradation rates and high chemical versatility, allowing for surface modifications, polymeric nanoparticles for constant rate release of therapeutic drugs, where surface functionalization can be designed to confer therapeutic targeting capabilities to improve targeting efficiency and sustained release of drugs. Arjun et al. (38) developed a genistein flavonoid (GEN)-containing FA receptor-targeted and poly(ethylene glycol)-fused poly nanoparticles (PLGA-PEG-FA NPs) that can deliver GEN-targeted to ovarian cancer cells. The PLGA-PEG-FA NPs can sustain the release of GEN, as well as increase the uptake of the drug by SKOV-3 cells, which has a better specific delivery potential compared to the nontargeted drug with better specific delivery potential compared to non-targeted drugs, and the conduct of this study provides an avenue for the development of GEN-targeted nanoparticles.

### 3.3 Ultrasound-targeted nanocarrier-mediated cavitation combination therapy

As for the nanodrug delivery system, ultrasound enhances the therapeutic effect through three main mechanisms: **(i)** triggering the drug release of nanoparticles at the target site; **(ii)** facilitating the penetration and distribution of nanoparticles and drugs in the tumor mesenchyme; and **(iii)** improving the efficiency of cellular uptake and internalization of drugs (39). Ultrasound can be used to improve drug delivery to tumors with low Enhanced Permeability and Retention (EPR). As ultrasound travels through the body, its rarefaction and compression cycles can interact with nanoparticles (NPs) in the circulating blood or NPs residing in the extracellular matrix, triggering the release of anticancer drugs at the target region (ROI). The interaction of ultrasound with NPs and cells is key to the optimization of drug delivery systems (DDS) for therapeutic purposes and combining the advantages of targeted ultrasound (TUS) and NPs delivery can achieve synergistic anti-cancer effects (40). In addition, ultrasound-mediated nano-DDS can help to overcome major limitations of current cancer treatments, such as low therapeutic efficiency and limited tissue penetration depth. The main drug-release mechanisms of polymeric NPs include diffusion, erosion, dissolution, partitioning, and swelling (41). Ultrasound can act on drug-carrying NPs that are sensitive to thermal or mechanical stimuli, causing them to melt (thermal effect) or rupture (mechanical effect) for drug release. Moreover, ultrasound also promotes the accumulation of drug-encapsulated nanoparticles in the target tissue. This facilitation is attributed to the mechanical energy carried by the ultrasound radiation, which physically propels the nanoparticles *in vivo*, and the physical propulsion enhances the delivery of nanoparticles to ultrasound-exposed tissues, leading to localized accumulation of nanoparticles and increased cellular uptake (42). It has been shown that ultrasound can cause polymer degradation to produce lower molecular weight fragments. This ultrasound-induced polymer breakdown is not random. In general, polymer chain fragmentation occurs more frequently in the middle of the chain and larger molecules are degraded more rapidly. Another remarkable feature of ultrasound-induced decomposition is that the decrease in molecular weight is due to the breaking of the weakest chemical bonds in the chain. Reich et al. (43) showed that the application of ultrasound significantly reduced the molecular weight of two polymers, including polylactic acid (PLA) and polylactic acid-glycolic acid copolymer (PLGA). Elhelf et al. (44) further demonstrated that ultrasound was effective in stimulating poly(lactic acid) (PLGA) degradation in the frequency range of 5 to 10 MHz. Therefore, the application of ultrasound may enable drug release. Recent studies have proposed that the high shear force field and hot spots during ultrasonic cavitation are the main drivers of polymer degradation and that shock waves and friction generate stresses at the surface of the polymer chain and in the inner coils during the collapse of the cavitation, which triggers the breakage of the macromolecular chain (45). Zhang et al. (46) discovered and synthesized multifunctional tumor-targeting poly(lactic acid)-glycolic acid copolymer (PLGA) nanoparticles (NPs-cRGD) to monitor therapeutic efficacy and overcome cisplatin resistance by dual-mode imaging, ultrasound has the advantage of facilitating the crossing of biological barriers and the rapid release of drugs and genes, and with the assistance of ultrasound and the targeting of NPs-cRGD, which can readily enter into the cells. NPs-cRGD escaped lysosomal degradation thereby protecting siBIRC5 from the enzyme, leading to down-regulation of BIRC5 expression and inhibition of BIRC5 protein in SKOV3-DDP.

## 4 Conclusions and prospects

In recent decades, attempts have been made to combine new technologies with traditional methods to fight cancer. The use of currently available chemotherapeutic agents (usually platinum-based drugs and paclitaxel preparations) in the conventional treatment of ovarian cancer remains challenging. Chemotherapy may be relapsed by the development of drug resistance, and ovarian cancer cells are known to be resistant to a variety of drugs, including cisplatin, carboplatin, and paclitaxel. Drug combinations can be selected to target multiple pathways to induce cell death to overcome resistance mechanisms. The formation of drug combinations into nanoparticles can promote sustained release, which can further enhance therapeutic efficacy. Even with emerging nano-drug delivery systems, their biological properties and safety still need to be validated in extensive clinical trials for subsequent dissemination. Due to the versatility of PLGAs, polymer-based nanoparticles are a particularly promising tool for the clinical translation of combination therapies with adjustable dosing regimens. Results in this area are encouraging as nanoparticle formulations have been found to increase *in vitro* cytotoxicity and reduce *in vivo* tumor volume compared to free drug formulations. The application of polymeric nanomaterials in the diagnosis and treatment of ovarian cancer has been increasing in recent years, Ramalingam et al. (47) prepared single crystal and irregularly shaped Fe2O3 NPs by a wet chemical process that exhibited significant *in vitro* cytotoxic activity against human metastatic ovarian cancer cell line (PA-1 cells) by increasing intracellular ROS levels, programmed cell death. Diagnostic and therapeutic agents can be encapsulated covalently conjugated or attached to PLGA nanoparticles with the help of a connector. PLGA-based nanoparticles provide a versatile platform for enhanced and effective diagnosis and treatment of cancer using a single activity or a combination of imaging, contrast, and therapeutic properties. Nanoparticles can minimize some of the unique issues with these drugs by ensuring stability and preserving their structure. In addition, nanoparticles offer clever therapeutics by enabling targeted delivery and controlled release.

The integration of nanoparticle engineering with computational models for predicting synergistic drug combinations and optimized dosing regimens represents a promising strategy to accelerate nanomedicine development, thereby improving ovarian cancer treatment. PLGA polymer nanoparticles show significant potential for cancer therapy and diagnostics. However, many studies show that there is still a large gap in the clinical translation of PLGA polymer nanoparticles in cancer therapy. We still face many challenges, such as optimizing ultrasound parameters and ultrasound response to nanoscale DDS to maximize the therapeutic efficacy and considering various biophysical effects in nanoscale DDS. However, we believe that with the in-depth study of genomics, genetics, biomedical engineering technology, and tumor-targeted therapy, PLGA polymer nanocarriers will have a broad application in the field of ovarian tumor-targeted therapy.

There is no doubt that nanomaterials are the most promising materials for future development in the biomedical field. Currently, there are fewer studies on GRPR (gastrin-releasing peptide receptor)-targeted drug-carrying nanoparticles applied to ovarian cancer, and relevant studies have shown that GRPR is highly expressed on ovarian cancer cells but not on normal ovarian tissue cells, Studies have shown that GRPR (gastrin-releasing peptide receptor) and NMBR (neuromodulin B receptor) are widely distributed in human ovarian cancer, and BRS-3 is found in stage IV tumors. As these are the three Suzuki peptide receptor subtypes in human ovarian cancer cell lines, we may consider using approaches based on ghrelin/GRP receptor antagonists or targeting ghrelin analogs to treat ovarian cancer (48). Meanwhile, the combination of GRPR with clinicopathological features such as tumor differentiation and ascites likewise suggest that it may be a promising target for determining the prognosis of ovarian cancer. So future studies can focus more on GRPR-targeted PLGA polymer drug-carrying nanoparticles combined with ultrasonic cavitation for ovarian cancer patients, and in this regard, also for ovarian cancer patients and improve their prognosis.
